# The Local Incidence of Infectious Diseases

**Published:** 1916-11-18

**Authors:** 


### THE LOCAL INCIDENCE OF INFECTIOUS DISEASES.

The Local Government Board's annual return, begun in 1911, giving statistics as to the local incidence of infectious diseases, has now reached its fifth issue. We should be able to test in some measure the value of these data, the collection and publication of which were made possible by the legislation, culminating in the general Act of 1898, requiring the notification of the principal infectious diseases. The diseases to which the reports relate comprise small-pox, scarlet fever, diphtheria, enteric fever, puerperal fever, and erysipelas. Of the other notifiable diseases plague and cholera are happily negligible quantities, whilst in respect to pulmonary tuberculosis the statistics as to local areas are not published for reasons set out in Dr. Newsholme's annual report for 1913-14. During the period under review small-pox has happily been in much the same position as cholera and plague. There remain scarlet fever and diphtheria as the two most prevalent diseases upon whose incidence sanitary conditions exert the greatest influence.

It is somewhat disconcerting to find that, taking the country as a whole, both diseases show an increase, not a decline. In England in 1911 scarlet-fever cases numbered 104,617; in 1915 they numbered 127,086. The comparative diphtheria figures are 47,147 and 53,597. The adverse difference as regards diphtheria is very much smaller, but is still sufficiently serious in view of the complacent assumption so often made that our social and hygienic conditions are steadily improving all the time. The more efficient carrying out of the law as to notification may partly explain the figures, but when full allowance has been made for this factor they cannot be said to be reassuring. The cause of such retrogression as is here revealed, whatever it may be, is evidently of national significance, but these returns have their distinctive value as a barometer of local conditions. Let us pass on, therefore, to consider them in this light. In 1911 scarlet fever was most prevalent in the counties of Monmouth, Northamptonshire (Soke of Peterborough), Leicester, Warwick, Cambridge, and Glamorgan. Of the county boroughs Coventry shares the same unenviable distinction with Stoke-on-Trent, Barrow-in-Furness, Leicester, and St. Helens. Turning to the figures for 1915, it is gratifying to find that with three exceptions all these black sheep have become less black. The three backsliders are the county of Monmouth, whose rate has further increased by almost half as much again, the county of Glamorgan and the borough of St. Helens, which both show further slight increases. In respect to diphtheria, among the counties, Flint, the East and North Ridings of Yorkshire, Hereford, the Isle of Wight, and Radnor had an undesired pre-eminence in 1911; and among the boroughs, Canterbury, Derby, Stoke-on-Trent, Reading, Croydon and Southampton. The comparison with 1915 is less satisfactory, two of the counties and three of the boroughs having gone from bad to worse.

Another side of the picture is shown by comparing the "best record" of 1911 with the figures for the same place in 1915. In the former year the boroughs of Tynemouth, Dudley, Oxford, Canterbury, West Bromwich, and Bournemouth, and the counties of Suffolk (East), Lincoln (Holland), Devon, Montgomery, Pembroke, and Anglesey had the lowest rates. Generally speaking, these positions of honour were maintained in 1915, but there were three relapses, Dudley, Canterbury, and Tynemouth. With respect to diphtheria, the lowest borough rates were those of Bournemouth, Wolverhampton, Rochdale, Dudley, Walsall, and West Bromwich, and the lowest county rates those of Lincoln (Holland), Anglesey, Buckingham, West Suffolk, Huntingdon, and Oxford. In Rochdale alone has there been any serious retrogression.

What is the moral to be drawn from these facts? To answer this question fully would require an inquiry into local conditions and circumstances. We think it may be safely assumed that this publication of good and bad marks has had its effect upon the public health authorities concerned. A bad position in the class has stimulated them to exertion until a better one has been secured. Conversely, but more exceptionally, a good position may have induced false confidence and foolish slackness, with the result that it has speedily been lost. To what extent the local incidence of infectious diseases is affected by such factors as varying efficiency in the system of notification, or of isolating patients, on the one hand, or sanitary conditions as regards housing, water-supply, etc., on the other, it is impossible to determine without local knowledge. But it is quite clear that from these annual returns such factors are of more importance than variations in social condition as between different classes of society.

